# An extended Weight Kernel Density Estimation model forecasts COVID-19 onset risk and identifies spatiotemporal variations of lockdown effects in China

**DOI:** 10.1038/s42003-021-01677-2

**Published:** 2021-01-25

**Authors:** Wenzhong Shi, Chengzhuo Tong, Anshu Zhang, Bin Wang, Zhicheng Shi, Yepeng Yao, Peng Jia

**Affiliations:** 1 Smart Cities Research Institute, Department of Land Surveying and Geo-Informatics, The Hong Kong Polytechnic University, 999077 Hong Kong, China; 2 College of Oceanography and Space Informatics, China University of Petroleum, 266580 Qingdao, China; 3 Research Institute for Smart Cities, School of Architecture and Urban Planning, Shenzhen University, 518060 Shenzhen, China; 4 International Institute of Spatial Lifecourse Epidemiology (ISLE), 999077 Hong Kong, China

**Keywords:** Risk factors, Infectious diseases

## Abstract

It is important to forecast the risk of COVID-19 symptom onset and thereby evaluate how effectively the city lockdown measure could reduce this risk. This study is a first comprehensive, high-resolution investigation of spatiotemporal heterogeneities on the effect of the Wuhan lockdown on the risk of COVID-19 symptom onset in 347 Chinese cities. An extended Weight Kernel Density Estimation model was developed to predict the COVID-19 onset risk under two scenarios (i.e., with and without the Wuhan lockdown). The Wuhan lockdown, compared with the scenario without lockdown implementation, in general, delayed the arrival of the COVID-19 onset risk peak for 1–2 days and lowered risk peak values among all cities. The decrease of the onset risk attributed to the lockdown was more than 8% in over 40% of Chinese cities, and up to 21.3% in some cities. Lockdown was the most effective in areas with medium risk before lockdown.

## Abstract (web summary)

Wenzhong Shi et al. propose an extended Weight Kernel Density Estimation model to predict the COVID-19 onset risk, with and without the Wuhan lockdown, and corresponding symptom onset and spatial heterogeneity in 347 Chinese cities. The authors find that the lockdown delayed COVID-19 peak onset by 1–2 days and decreased onset risk by up to 21%.

## Introduction

Emerging infectious diseases, such as the coronavirus disease 2019 (COVID-19), have become global challenges for the public health sector^1,2^. This new virus is highly contagious and can be transmitted through respiratory droplets or physical contact^3,4^. As of 8 April 2020, 1,353,361 people worldwide have been identified as being infected by COVID-19^5^. Confirmed cases have appeared in 213 countries or regions^5^, and currently, the number continues to rapidly increase. With the fast development of modern transportation, people are currently able to travel on an accelerating scale and speed, which could make pathogens spread more easily, hence greatly aggravating the disease transmission. To reduce the spread of COVID-19, China, from 23 January 2020, imposed lockdown measures in Wuhan, China’s epicentre of the COVID-19 pandemic at that time. The Wuhan lockdown basically prevented anyone from entering or leaving Wuhan by any means of transportation, as well as limiting local movements of residents to only critical activities, including medical treatment, epidemic prevention and security operations^6^. From around 10 February 2020, just 18 days after the implementation of the lockdown, an obvious drop in daily new COVID-19 infections was witnessed in all of China’s provinces except for Hubei. Due to the early decreases in new infections, the relatively limited healthcare resources, across China, could be devoted to both COVID-19 and also the non-COVID-19 patients in the greatest need. Thus, when necessary, such as at the height of the pandemic, healthcare resources were sufficient to be reallocated to COVID-19 patients in Wuhan, hence contributing to the enablement of the whole country recovering from the epidemic more rapidly. By the end of the Wuhan lockdown on 8 April 2020, 77,838 (~93%) confirmed cases across China have recovered^7^.

Lockdown increasingly appears to have been necessary to enable a country to curb the current escalating pandemic, thus it is of prime importance to provide scientific evidence regarding the exact effect of lockdown measures and to what extent such measures could bring COVID-19 under control^8^. It is important to note that early effort has been made in this direction, however the findings are mixed. For example, Yang et al.^9^ showed that, if Wuhan had been locked down 5 days later, the cumulative number of COVID-19 infections from 23 January to 24 April 2020 would have tripled in China. Wu et al.^10^ predicted that, even with the lockdown implemented in Wuhan, many other major cities in China would still undergo local outbreaks, with the similar exponential growth of infections seen in Wuhan.

A recent study estimated that the Wuhan lockdown was associated with the later appearance of the reported COVID-19 cases in other cities by 2.91 days (95% CI: 2.54–3.29)^11^. However, most existing studies have used mathematical models for infectious disease transmission that rely on theoretical epidemiological parameters for making prediction. Such mathematical models include, for example, Susceptible-Infected models, Susceptible-Infectious-Recovered models, Susceptible-Exposed-Infectious-Recovered models and Logistic models. In these models, the spatial relationships among cities typically have been downplayed. Facing new infectious diseases (e.g., COVID-19) with limited prior knowledge of their features and also limited associated comparability with previous diseases, there has been much uncertainty in setting the theoretical parameters and assumptions of mathematical prediction models^12–15^. Knowledge uncertainty such as this could possibly have led to the highly mixed and uncertain conclusions among the above, existing studies. Moreover, most existing models make predictions based on the diagnosis date (i.e., the date on which the cases are confirmed) in historical data. Also, these models make predictions regarding future occurrences of confirmed COVID-19 cases. The estimated diagnosis date is usually later than the date of symptom onset. Importantly, it has been found that COVID-19 patients are at their most infectious during the first week after the symptom onset^16,17^. Thus, if using findings based purely on the COVID-19 diagnosis dates, as opposed to the date of symptom onset, the key period for enabling the prevention of further COVID-19 infections may be missed.

A common way for infectious diseases to spread is by the spatial movements of dynamic hosts and/or vectors^18–20^. Thus, for exploring the risk progression of new infectious diseases such as COVID-19, the adoption of appropriate data-driven spatial models has been considered a minimum requirement. Further, such models could better enable the evaluation of the effects of epidemic containment measures (e.g., the Wuhan lockdown)^21^. The Weight Kernel Density Estimation (WKDE) model is one such model. This model conducts retrospective analyses, on the basis of location information, in order to estimate the dates of infection of the confirmed cases. By this means, the place-specific risk of infection caused by spatial movements of infected people is then predicted^22^. Such spatial, data-driven models could alleviate the reliance on theoretical assumptions and parameters, and thereby, in the contexts of new infectious diseases, have a reasonable potential of making robust and place-specific predictions. The WKDE model, however, did not consider the effects of such modern factors as travelling by highway or express trains. By such means, journey speed could elevate disease transfer speed, at a greater rate, compared to that of traditional travel modes, between the areas of interest. To achieve a more accurate prediction, it is necessary to use dynamic mobility data to capture such modern factors, and moreover, to improve existing spatial models, so as to incorporate such big data into prediction.

This study aims, for the first time to the best of our knowledge, to conduct a comprehensive and high-resolution investigation into the spatiotemporally heterogeneous effects of the Wuhan lockdown on the risk of the COVID-19 symptom onset in 347 Chinese cities. The 347 Chinese cities include all prefecture-level cities except for Sansha City which is free of COVID-19 infections, four municipalities directly under the central government (Beijing, Shanghai, Chongqing and Tianjin), and two special administrative regions (Hong Kong and Macau). To achieve this goal, an extended WKDE model was developed, on the basis of the original WKDE model, to forecast the risk of COVID-19 symptom onset under two scenarios: (a) with the Wuhan lockdown and its implications and, (b) without the Wuhan lockdown. The effects of the Wuhan lockdown on the risk of the COVID-19 onset at a high spatiotemporal resolution across China were then analysed by comparing the predicted onset risk, based on each of these two scenarios. To predict the risk of the COVID-19 onset with the consideration of the effect of high-speed travel, the extended WKDE model has incorporated inter-city human mobility data to calibrate traditional spatial relationships among cities. The analysis was based on a spatiotemporal dataset of 40,486 confirmed COVID-19 cases in China during the period from the 31 December 2019 to 2 March 2020 (the 40th day after the Wuhan lockdown). Of these cases, 1189 had reported dates of symptom onset recorded^23^. The dates of symptom onset for the remaining 39,297 cases were estimated by an established statistical method^24^. Both the daily Wuhan out-migration to all other cities and the percentage of Wuhan migrants to every other city were calculated to indicate inter-city human movements in the extended WKDE model.

## Results

### Prediction accuracy of the extended WKDE model

In this study, the accuracy of the predicted risk of symptom onset was evaluated daily. The prediction accuracy was defined as the percentage of confirmed cases reported in areas where the predicted risk of symptom onset was >0.8. For example, if 100 cases were confirmed in the entire study area on the date for which the prediction is made, and out of which 77 cases occurred in the areas with predicted onset risk over 0.8, then the prediction accuracy on that date was 77%. This definition of the prediction accuracy followed the idea of hit rate^25,26^, the popular indicator for the prediction accuracy for the Kernel Density Estimation model on which the original and extended WKDE models were based.

**Fig. 1:**
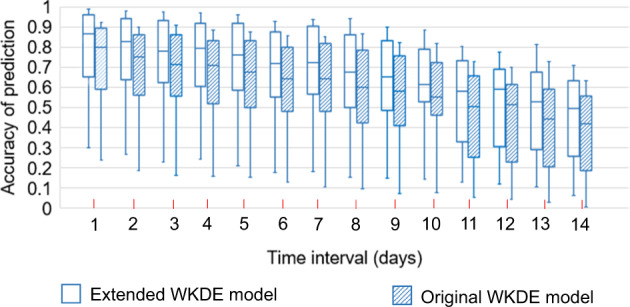
Accuracy of the predicted risk of COVID-19 symptom onset by the extended and original Weight Kernel Density Estimation (WKDE) models, under the scenario of Wuhan lockdown. The predicted onset risk is a standardised value between 0 and 1, indicating risk relative to the highest predicted risk among all locations on the date for which the risk of symptom onset is predicted, hereafter termed “the prediction date” (see Methods section for further detail). The prediction accuracy is defined as the percentage of the confirmed cases in the areas in which the predicted onset risk was >0.8^22^ on the prediction date. The time interval denotes the period between the base date and the date of prediction. The horizontal line in the box denotes the median, while the lower and upper edges of the box represent the respective first and third quartiles. The lines emanating from the box upwards and downwards represent the respective maximum and minimum values.

The extended WKDE model resulted in prediction accuracy of over 70% in the first week after the base day. The extended WKDE model achieved higher accuracy than the original WKDE model, judged by both visual observation (Fig. 1) and statistical evaluation (Table 1). Such an outperformance should be attributed to the incorporation of human mobility, the key difference between the original and extended WKDE models. The prediction accuracy during the second week after the base day, as possibly could be expected, was lower due to the accumulation of prediction errors over time.

**Table 1:** Result of two-sample *T*-test on the prediction accuracy on all dates of the extended and original WKDE models.

No. of days between base date and date of prediction		1	2	3	4	5	6	7	8	9	10	11	12	13	14
Accuracy of extended WKDE	Mean	0.773	0.759	0.745	0.734	0.725	0.700	0.706	0.673	0.655	0.593	0.549	0.512	0.512	0.459
	Variance	0.084	0.075	0.071	0.065	0.060	0.059	0.059	0.056	0.055	0.057	0.052	0.049	0.045	0.043
Accuracy of original WKDE	Mean	0.679	0.665	0.657	0.650	0.644	0.616	0.625	0.590	0.574	0.515	0.471	0.435	0.428	0.385
	Variance	0.082	0.074	0.070	0.063	0.055	0.057	0.055	0.056	0.053	0.056	0.051	0.050	0.045	0.042
*T*		1.988	2.106	2.016	2.031	2.069	2.119	2.099	2.136	2.129	2.017	2.096	2.105	2.438	2.210
*P*(*H*_0_)		0.049	0.037	0.046	0.044	0.040	0.036	0.038	0.034	0.035	0.046	0.038	0.037	0.016	0.029

The null hypothesis was *H*_0_: *μ*(*accuracy*_extended_) ≤ *μ*(*accuracy*_original_), i.e., the extended WKDE model is not more accurate than the original WKDE model. At the significance level of 0.05, *H*_0_ was rejected consistently for the prediction accuracy with 1–14 days between the base date and the prediction date, thus showing that the extended WKDE model has a statistically significant higher accuracy. *N* = 75 samples on every date were use in each two-sample *T*-test.

The extended WKDE model was found to be able to correctly identify a relatively small number of cities in which most confirmed cases were anticipated in the near future and indeed confirmed. This success could help enable targeted epidemic prevention efforts. During the entire study period, the average number of confirmed cases in cities with predicted risk of over 0.8 was almost 20 times greater than those in cities with predicted risk between 0.6 and 0.8 (Table 2). Even when the overall morbidity risk in China was the most severe, only 115 (33.1%) of the 347 Chinese cities had onset risk >0.8 (30 January 2020 in Table 3). During most of the study period, ~15% or fewer cities had onset risk >0.8. Indeed, the average number of confirmed cases per city increased significantly during each higher interval of predicted onset risk (Table 2), thus illustrating that, in general, the model can also correctly predict the relative onset risk among mid- and low-risk cities.

**Table 2:** The relationship between the predicted risk of COVID-19 symptom onset shown by the extended WKDE model and the average number of confirmed cases in cities.

Onset risk value in cities	Average number of confirmed cases in cities
0–0.2	0.0836
0.2–0.4	0.416
0.4–0.6	0.896
0.6–0.8	2.16
0.8–1	41.81

**Table 3:** The number of cities, at different onset risk levels, under two scenarios (i.e. with and without Wuhan lockdown measure) on the dates corresponding to Fig. 2e–j.

No. of cities	Onset risk					Wilcoxon signed-rank test, *P* (*H*_*0*_)
	0–0.2	0.2–0.4	0.4–0.6	0.6–0.8	>0.8	
25 January 2020 Lockdown	183	43	48	56	17	1.180 × 10^−58^
Non-lockdown	124	58	26	98	41	
30 January 2020 Lockdown	126	40	41	25	115	2.009 × 10^−37^
Non-lockdown	101	35	23	28	160	
5 February 2020 Lockdown	178	67	51	26	25	1.076 × 10^−57^
Non-lockdown	126	49	39	86	47	

The rightmost column shows the *P* values of the Wilcoxon signed-rank test on paired onset risk values of a city under two scenarios on the same date. The null hypothesis of this test, *H*_0_, is that the median difference between the onset risk values under lockdown and non-lockdown scenarios is zero. At a normal significance level, *H*_0_ could be rejected, thus showing that the onset risk under the non-lockdown scenario is statistically significantly higher. *N* = 347 samples on every date were use in each Wilcoxon signed-rank test.

### Spatiotemporal evolution of the risk of COVID-19 symptom onset

**Fig. 2:**
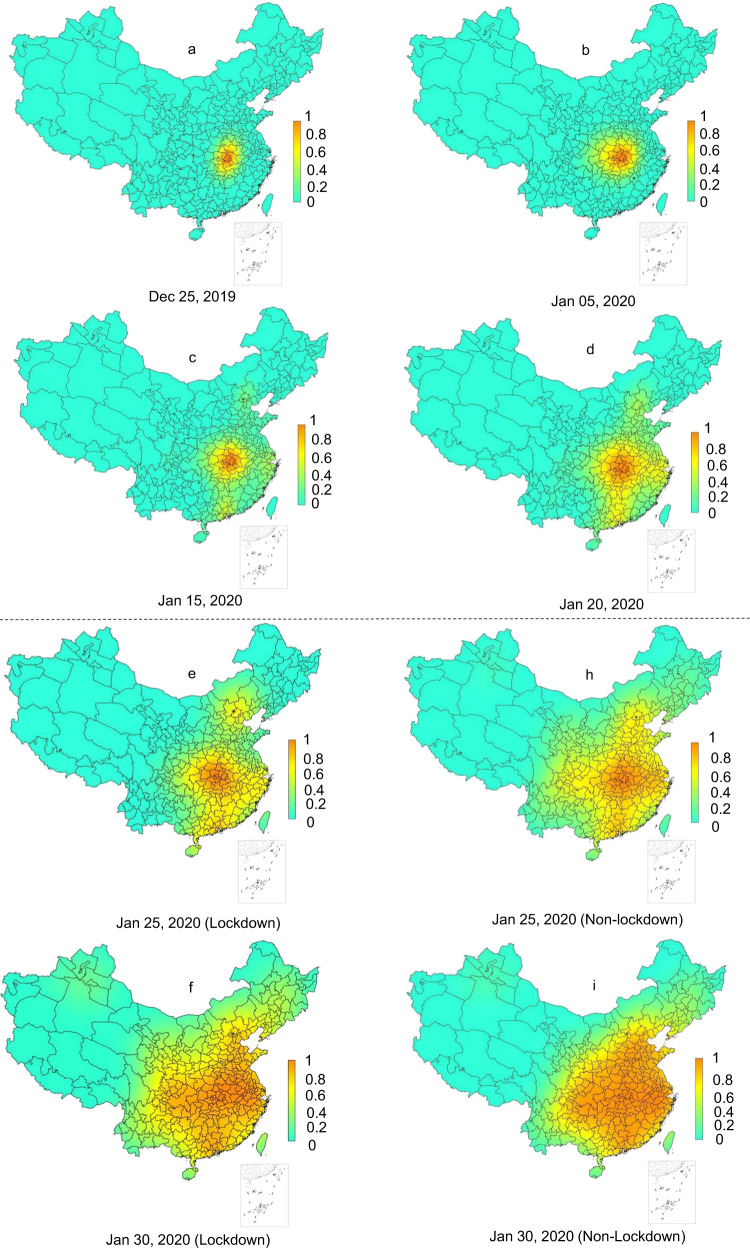

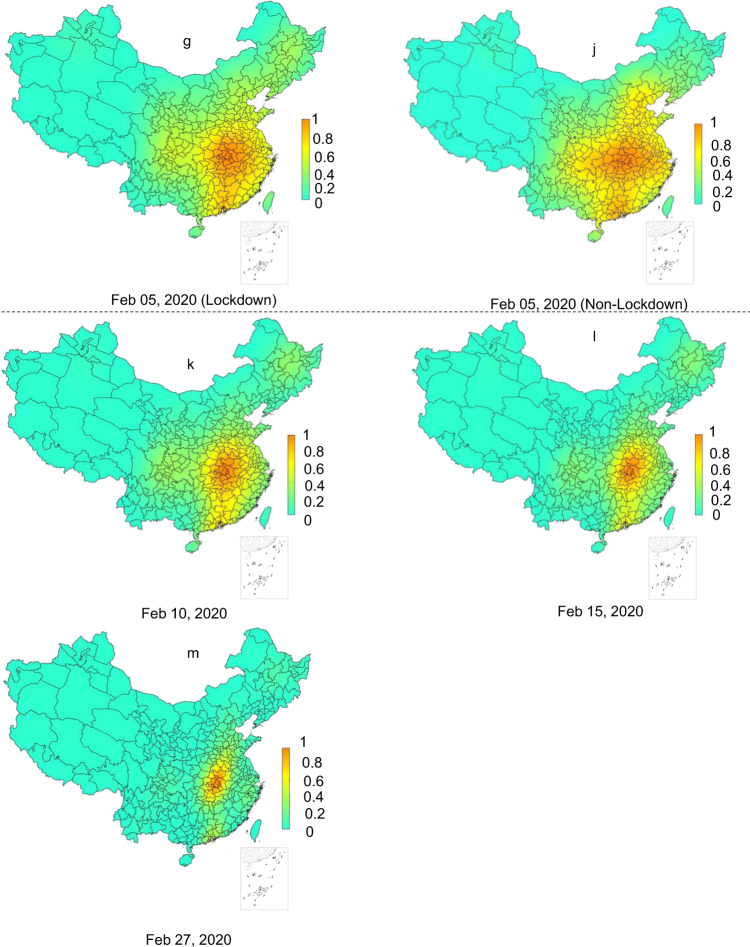
Predicted risk of COVID-19 symptom onset across 347 Chinese cities. **a**–**d** The predicted onset risk before the date of Wuhan lockdown. **e**–**m** The predicted onset risk after the date of Wuhan lockdown under two scenarios, i.e., with (**e**–**g**, **k**–**m**) and without (**h**–**j**) Wuhan lockdown. The predicted COVID-19 symptoms onset risk were generated by the extended WKDE model, by using historical confirmed cases and inter-city human mobility data. The historical confirmed cases data included the locations at which these cases had a period of study prior to the diagnosis. The predictions under two scenarios were made by differing the human mobility intensity from Wuhan to other cities after the date of the Wuhan lockdown. Under the lockdown scenario, the outward human flows from Wuhan were regarded as not increasing the onset risk of other cities, due to the dramatic decrease in human mobility intensity, together with strict quarantine measures. Under the non-lockdown scenario, the human mobility intensity was estimated based on that of the corresponding time period in 2019 (see Methods section for details).

The risk of the COVID-19 symptom onset during the study period, under the scenario of the imposed Wuhan lockdown (i.e., the reality) was first estimated by the extended WKDE model. The spatiotemporal variations of the estimated risk are described as follows. On 25th December 2019, areas with the risk of COVID-19 symptom onset >0.8 were only in Wuhan and the surrounding cities, most of which were largely in eastern Hubei Province; the onset risk in 96.8% of the areas in China was <0.2 (Fig. 2a). The influences of human mobility on the onset risk were not apparent until approximately one week before the Wuhan lockdown, during which time, cities, such as Chongqing (west), Beijing (north) and Guangzhou (south) which received a large number of passengers from Wuhan, started to present a higher position of onset risk (Fig. 2c). Approximately 1 week after the Wuhan lockdown, the overall onset risk in China reached the highest level (Fig. 2f). The onset risk then steadily decreased over the whole area outside Hubei Province. The onset risk following two weeks of the Wuhan lockdown (Fig. 2g) dropped below 0.6 in 64.3% of cities outside Hubei Province. The risk following three weeks of the lockdown (Fig. 2k) dropped below 0.6 in 81.6% of cities outside Hubei. By the end of the fifth week following the Wuhan lockdown, the areas with the onset risk >0.8 shrank to Wuhan and the surrounding cities in Hubei (Fig. 2m).

### Spatiotemporal patterns of effects of the Wuhan lockdown regarding COVID-19 onset risk

**Fig. 3:**
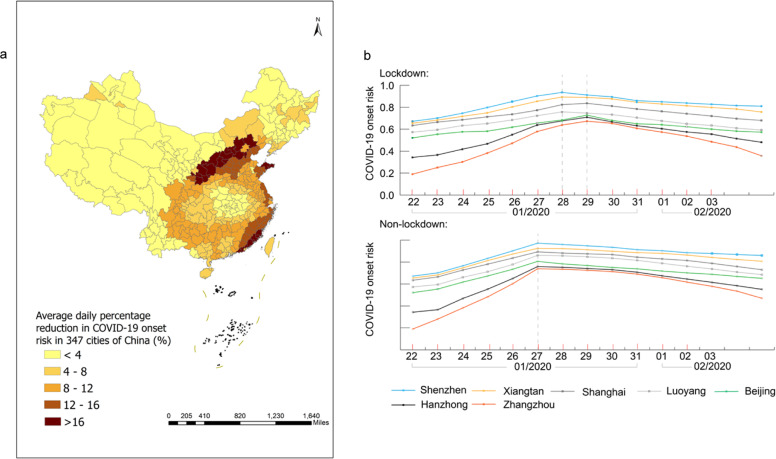
The risk of COVID-19 symptom onset under two scenarios (i.e., with and without Wuhan lockdown measure) from 24 January to 5 February 2020. **a** Average daily percentage reduction in the onset risk in 347 Chinese cities in the lockdown scenario, compared with the non-lockdown scenario. **b** The onset risk under two scenarios in seven selected cities. The plotted values were computed from the predicted risk of COVID-19 symptom onset under the two scenarios resultant from the extended WKDE model, by using historical confirmed cases and inter-city human mobility data. Under the lockdown scenario, the outward human flows from Wuhan were regarded as not increasing the onset risk of other cities, due to the dramatic decrease in human mobility intensity, together with strict quarantine measures. Under the non-lockdown scenario, the human mobility intensity was estimated based on that of the corresponding time period in 2019 (see Methods section for details).

The risk of the COVID-19 symptom onset was predicted under two scenarios by the extended WKDE model, that is, with and without the Wuhan lockdown. The effects of the Wuhan lockdown on the risk of the COVID-19 onset were then evaluated based on the predicted risk under these two scenarios. Compared to the COVID-19 onset risk under the lockdown scenario (Fig. 2e–g), the onset risk under the non-lockdown scenario, on the same date, was significantly higher (Fig. 2h–j; Table 3). For example, in Guangdong Province, which typically receives large Wuhan migration, from 24 January to 5 February 2020, onset risk values in all the 21 cities had been reduced by 7.56% on average under the lockdown scenario, compared with those of the non-lockdown scenario. Around the time when the onset risk reached the peak (i.e., at that moment, the overall onset risk of COVID-19 in 347 cities in China reached the maximum) (Fig. 2i), the areas with onset risk >0.8 would have expanded to include most Chinese cities, except for those in the north and west, which had low inter-city population flows from Wuhan. In all 347 cities, the risk values of the COVID-19 onset under the lockdown scenario were, indeed, lower than those under the non-lockdown scenario. The decrease in the onset risk attributed to the Wuhan lockdown achieved up to 21.3% throughout all cities. The decrease in the onset risk was more than 8%, 12% and 16% in 146, 58 and 28 cities, respectively (Fig. 3a). Note that these decreases are the most conservative estimates, as the predicted onset risk, under the non-lockdown scenario, was made based on the confirmed cases under the lockdown scenario. Most, if not all cities without the Wuhan lockdown, in theory, would have been likely to have more than the currently recorded cases. Therefore, the actual effect of the Wuhan lockdown on subsequent risk decrease of COVID-19 symptom onset should undoubtedly have been greater.

A daily comparison of the predicted onset risk under the lockdown and non-lockdown scenarios reflect the contributions by the Wuhan lockdown in three aspects: a constant lower daily onset risk after the lockdown, the delayed arrival of peaks of the daily onset risk by 1–2 days, and the subsequent lower peak risks. These three contributions have been observed in megacities such as Shanghai, Beijing and Shenzhen; in major-size cities such as Luoyang (in Henan Province); in medium-size cities such as Xiangtan (Hunan) and Zhangzhou (Fujian); and in minor-size cities such as Hanzhong (Shaanxi; Fig. 3b). The selection criteria of the above seven cities for this study is detailed in Supplementary Table 1. On 25 January, 30 January and 5 February 2020, the number of cities with a predicted risk of symptom onset >0.8 under the non-lockdown scenario was consistently much larger than that under the lockdown scenario (Table 3). Moreover, by approximately 3 weeks after the Wuhan lockdown, of all cities outside Hubei, in the 285 cities which had had peak risk values of above 0.4, the risk of symptom onset had decreased by 21.1% to 78.9%, relative to the corresponding peak risk values. In the 27 cities with peak risk values of between 0.2 and 0.4, risk decrease ranged from 15.7% to 62.4%. In the 18 cities with peak risk values of below 0.2, risk decrease ranged from 0.2% to 5.1% (Fig. 3a).

**Fig. 4:**
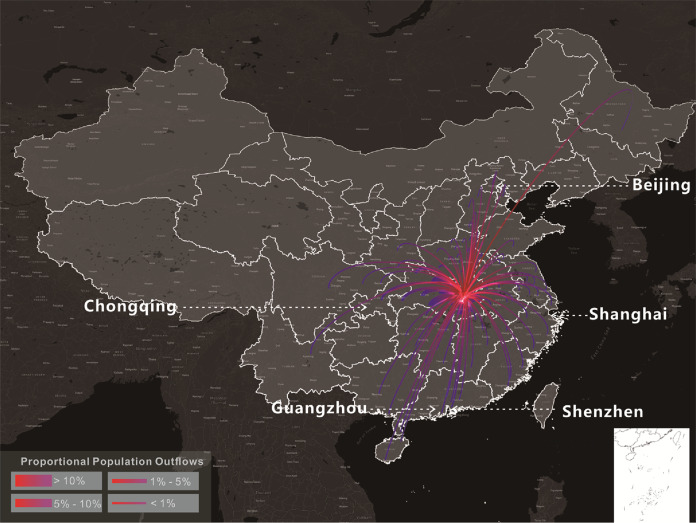
The percentage outflows of migrants from Wuhan to 100 cities in China which received the largest numbers of Wuhan migrants, on 8 December 2019–23 January 2020. The lowest percentage of Wuhan migrants received by any of the 100 cities was 0.02%. This figure is based on the massive positioning service data on the Baidu Map platform, available on https://qianxi.baidu.com/^38^. Several hotspot destinations of the Wuhan migrants are labelled in this figure. Lines with gradient colours represent the distance from the origin (Wuhan, shown in red) to the 100 cities.

The reduction of the onset risks in different cities attributed to the Wuhan lockdown were heterogeneous (Fig. 3a) and related to two factors: the intensity of human mobility from Wuhan to individual other cities in normal days, and the level of existing onset risk in individual cities by the date of the Wuhan lockdown. Most cities in western and northeastern China showed a small reduction (<4%) of the onset of risk attributed to the Wuhan lockdown (Fig. 3a). The human flows from Wuhan to these cities was generally quite small in normal days, with very few cities receiving >0.02% out-migrants from Wuhan (Fig. 4). In the seven selected cities which were deemed representative of all Chinese cities in terms of the geography and economic development, it was also observed that the higher the existing onset risk by the lockdown date, the smaller the percentage reduction in onset risk was attributed to the Wuhan lockdown (Fig. 3b). In the whole country, areas with a high percentage (>8%) of reduction of onset risk attributed to the Wuhan lockdown (Fig. 3b) largely coincided with areas with medium risk of onset (0.2–0.6) before the lockdown (Fig. 2d).

## Discussion

This study has provided data-driven evidence on the effects of the Wuhan lockdown measures on the risk of COVID-19 symptom onset in 347 Chinese cities in a high spatiotemporal resolution. That is, changing patterns of the risk of COVID-19 symptom onset has been demonstrated at city level on a daily basis. Specifically, the Wuhan lockdown has lowered the peak of the daily onset risk, delayed the arrival of the peak of onset risk for 1–2 days in other cities, and decreased the onset risk in those cities after the Wuhan lockdown on, compared to the scenario without the lockdown. The Wuhan lockdown was also found to reduce the infection risk by imported cases from Wuhan in other cities within one week after the lockdown had been imposed. This situation varied across cities: cities receiving larger volume of Wuhan migrants in normal days, in general, could avoid larger risk of symptom onset. Furthermore, the Wuhan lockdown was found to be most effective in reducing the onset risk in areas with medium risks prior to lockdown.

Findings from this study have important and unique implications for public health and especially epidemic response. They would serve as strong place-specific evidence regarding the effectiveness and efficiency of the city lockdown measures in reducing the COVID-19 onset risk. Results presented in this study can also be supported by those from some recent studies, which concluded from the results of Susceptible-Exposed-Infectious-Recovered model and global epidemic and mobility model that the Wuhan lockdown delayed the arrival of COVID-19 in other cities by 2.91 days^11^ and 3–5 days^27^, respectively. However, the delayed arrival presented in those studies referred to the ‘delayed reporting’, as only the reported dates of confirmed cases were used. This current study has predicted the risk of symptom onset, which typically occurs days before the diagnosis and reporting of the cases, and corresponds to the time at which the patients are the most infectious^16,17^. Such predictions could inform public health agencies ahead of catastrophic spread and hence guide local epidemic control and subsequently precise prevention efforts. Beyond early warnings, this study sheds further light on spatiotemporal heterogeneities in the onset risk, which would enable precision disease control and prevention. For example, by using the extended WKDE model, the exact areas of high risk of symptom onset and the level of risk in the near future could be predicted, thus the decisions concerning precise interventions could be better enabled.

This study does have some limitations. Firstly, only 40,486 confirmed cases with community-level locations were used rather than all 75,465 confirmed cases revealed during the study period in China. The incompleteness of such exposure history collected from confirmed cases could affect the results. Such effects, however, are considered limited toward our study aim, which focuses more on the areas outside Hubei Province. Cases without detailed locations mainly took place in severely affected cities, such as Wuhan. In those cities, people were intensively engaged in treatments and quarantine, and thus had limited capacity of committing to such epidemiological data collection efforts. Secondly, the method of estimating dates of symptom onset used in this study, although officially documented^24^, may still be subject to uncertainties, which needs to be further improved in the future. Thirdly, COVID-19 infectiousness and susceptibility in different populations were not considered in the current model, due to incomplete demographical information. Although this is considered acceptable as the travel and contact history has been playing a major role in the COVID-19 infection, more detailed demographical information and confirmed findings on varying infectiousness and susceptibility from clinical studies would further improve the quality of the model. Lastly, the current model considers only out-migrants from Wuhan without including travel flows between other cities, although the latter has been largely minimised within the study period, due to a high tendency to avoid COVID-19, and lockdown measures implemented to a different extent across China. Due to lack of relevant data, the current model did not consider the population who migrated from Wuhan, either, after the lockdown was announced at early morning on 23 January 2020 and before it took effect at 10.00 a.m. on the same day.

Despite the limitations listed above, this study has presented a high-resolution spatiotemporal prediction leading to important findings, thanks to the detailed publicly available information gathered from anonymous cases. Transparent reporting of travel and contact history of such a large number of anonymous infected cases has been realised in China, thus opening an avenue in the era of big data, for more advanced models to refine results from mathematical prediction models. It also enables and encourages multiple stakeholders outside the public health sector to be involved in collaborative control and prevention efforts to contribute to the curbing of increasingly more frequent and complex epidemics in the future^28,29^.

The high spatiotemporal resolution evidence from this study would be necessary for lockdown decision-making not only in all countries undergoing the first wave of infections, but in African and Latin American countries that will inevitably be hit by the next wave of infections^30^. More importantly, the extended WKDE model can be automatically fed by near real-time spatiotemporal surveillance data from multiple sources to make robust, timely predictions, which is difficult to be realised in traditional approaches. The model can also be flexibly extended by more available data, such as the effects of non-pharmaceutical interventions (NPIs) taken by various cities, to further calibrate the prediction. Such predictions would be a core component of epidemic early warning systems regarding the prevention of the future epidemics^31,32^.

## Methods

### Data sources

Spatiotemporal data of 40,486 confirmed COVID-19 cases in China from the 31 December 2019 to 2 March 2020 (the 40th day after Wuhan lockdown) were collected in this study. These cases were mainly collected from official reports produced by provincial and municipal health commissions^33–36^. For places where the official reported confirmed cases were incomplete, especially at the beginning stage of the epidemic, supplementary confirmed cases were collected from public media, such as the People’s Daily^37^, Tencent Health^38^ and Baidu Map^39^. These cases had available reporting dates, and self-reported locations at the community level where they had a period of stay prior to diagnosis. Among the collected cases, 1,189 cases had reported dates of symptom onset, with the earliest symptom onset dated on 8^th^ December 2019^23^. Among the total of 75,465 confirmed cases during this period^23^, the 40,486 cases were all the cases with available community-level locations founded in the data collection process of this study.

Two human mobility parameters derived from massive positioning service data in China on the Baidu Map platform^40^ were obtained during January–March 2019 and 2020. The first parameter was the daily Baidu out-migration index for Wuhan. This represented the magnitude of the daily population leaving Wuhan and travelling to all other cities. The second parameter was the percentage of Wuhan migrants travelling to every other city (Fig. 4). Also obtained were the daily passenger loads of the flights, railways and bus services from Wuhan to every other city from December 2019 to March 2020^41–43^.

### Daily migration

Two indicators of daily migration during the study period were calculated. They include: the daily Wuhan out-migration to all other cities, and the daily percentage of Wuhan migrants to every other city. The indicator values from 1 January 2020 were directly obtained from the Baidu migration data. However, the Baidu migration data were unavailable for December 2019. Therefore, both indicators for December 2019 were calculated based on Baidu data in early January 2020 and the daily passenger load data:

- The daily Wuhan out-migration for December 2019 was calculated as follows. First, the average of the ratios on each day in early January 2020 between the daily Baidu out-migration index and the daily passenger loads was obtained. Second, the daily Wuhan out-migration was computed by multiplying that average ratio by the daily passenger loads leaving Wuhan to other cities in December 2019. The result of this calculation was produced by assuming that the daily passenger load factor was stable during both December 2019 and early January 2020.
- The daily percentage of Wuhan migrants to every other city, during December 2019, was estimated as the daily percentage of passenger load departing from Wuhan to every other city. This method was also used to calculate the daily percentage of migrants in 2020 from Wuhan to those cities receiving a considerably small number of migrants from Wuhan. It is of note that in such cities, the Baidu migration data were suppressed from reporting for confidentiality reasons.

### Date of symptom onset for COVID-19 cases

**Fig. 5:**
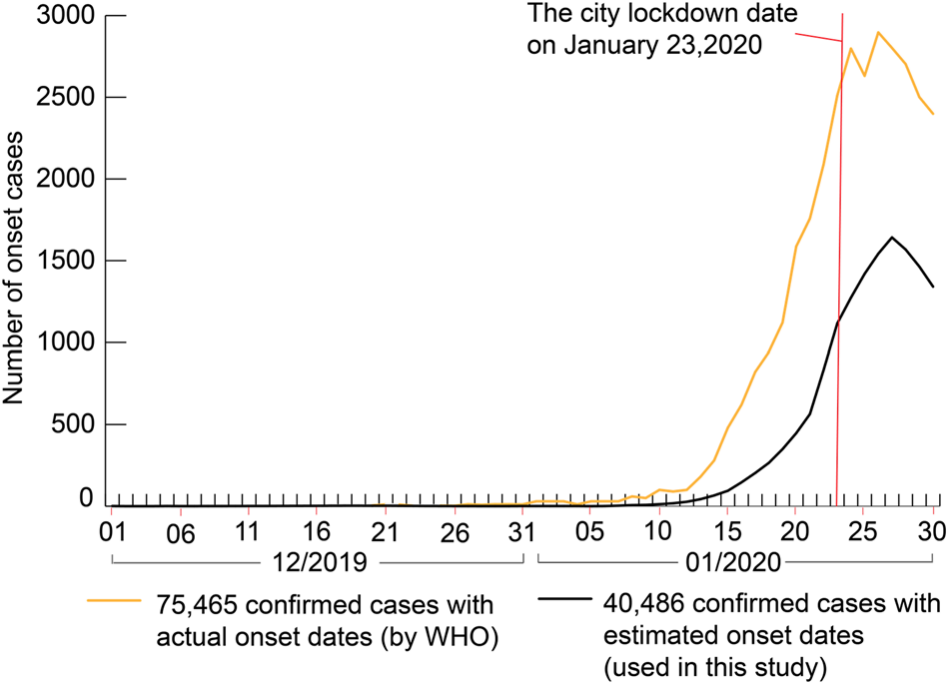
Distribution of the estimated dates of COVID-19 symptom onset among 40,486 confirmed cases used in this study, and of the actual dates of COVID-19 symptom onset among 75,465 confirmed cases reported by the World Health Organization. The available data on 40,486 confirmed cases in China from 31 December 2019 to 2 March 2020 were collected from official reports by provincial and municipal health commissions and public media. These cases had the dates on which they were reported, together with the community-level locations at which they had a period of stay prior to diagnosis. Of these cases, 1189 had reported dates of symptom onset. The statistical method proposed by Shi et al.^24^ was used to estimate the onset dates of the cases for which the onset dates were unavailable. The 75,465 confirmed cases were reported by the WHO^23^ and did not include the fine-scale location needed in this study.

An established statistical method proposed by Shi et al.^24^ was adopted to estimate the dates of symptom onset for the 39,297 confirmed cases which did not have reported onset dates. This method was based on an erlang distribution of the waiting time from the onset date to the diagnosis date of the confirmed cases, and had good performance in supporting the estimation of the onset date of confirmed cases in China. The method results in a probability *p*_waiting_(Δ*t*) that each confirmed case had a waiting time of Δ*t* day(s) between onset and diagnosis. Among *m* confirmed cases in the same city, and on the same day, a total of *m*∙*p*_waiting_(Δ*t*) confirmed cases were randomly selected and assigned a waiting time of Δ*t*. As a result, the distribution of the dates of symptom onset regarding the 40,486 confirmed cases used in this study is consistent with that of the actual dates of symptom onset of all 75,465 confirmed cases reported by the World Health Organization^23^ (WHO) (Fig. 5).

### The extended WKDE model for predicting the risk of COVID-19 onset

The model made the prediction by means of the following three steps:

*Step 1*: retrospective inference regarding the historical existence of the COVID-19 infection at each location, in which a confirmed case had a period of stay prior to diagnosis. The main aim of this step is to estimate the infection date of each confirmed case, given the date of symptom onset. From the infection date, that case had the risk of transmitting pathogens to others. The incubation period of each confirmed case from infection to symptom onset is modelled by following a Weibull distribution^44^.

1 $$p_{{\mathrm{incubation}}}\left( {{\mathrm{{\Delta}}}t} \right) = k\lambda ^{ - k}{\mathrm{{\Delta}}}t^{k - 1}{\mathrm{e}}^{ - ({\mathrm{{\Delta}}}t/\lambda )^k}$$

where *p*_incubation_(Δ*t*) denotes the probability that the incubation period of each confirmed case equals to Δ*t* days; *λ* and *k* denotes the mean and standard deviation of the incubation period, which, in this study, was assumed to be 5.2 and 2.8 days, respectively^45–47^. The natural exponential is denoted by e.

All days in this study period (8 December 2019 to 27 February 2020) are in the order denoted as *t*_1_, *t*_2_,… *t*_82_. The probability that each confirmed case was infected on a certain day and thus became infectious is:

2 $$P_{{\mathrm{infection}}}\left( {L,t_i} \right) = 1 - \mathop {\prod}\limits_{t_L > t_i} {\left( {1 - p_{{\mathrm{incubation}}}\left( {t_L - t_i} \right)} \right)} ^{n\left( {t_L} \right)}$$

where *P*_infection_(*L*, *t*_*i*_) denotes the probability that one confirmed case at location *L* was infected on day *t*_*i*_; *t*_*L*_ denotes the day of symptom onset for the confirmed case at location *L*; *n*(*t*_*L*_) is the number of onset cases at location *L* on day *t*_*L*_; *p*_incubation_(*t*_*L*_-*t*_*i*_) denotes the probability that the incubation period of the confirmed case is equal to (*t*_*L*_–*t*_*i*_) days.

*Step 2*: spatial extrapolation for inferring historical existence of COVID-19 infection in the whole study area. Let *L*_1_, *L*_2_,… *L*_*n*_ be the unique locations of all confirmed cases used for the prediction. The risk of infection at each random location is estimated as:

3 $$P_{{\mathrm{infection}}}\left( {S,ti} \right) = n\left( {t_i} \right)^{ - 1}\mathop {\sum}\limits_{j = 1}^{n(t_i)} {M\left( {S,t_i} \right)P_{{\mathrm{infection}}}( {L_j,t_i} )K_{\mathrm{h}}( {S - L_j} )}$$

where *P*_infection_(*S*, *t*_*i*_) denotes the probability that any infected person visited a random location *S* on day *t*_*i*_, and posed the risk of infection to others nearby; *L*_*j*_ denotes the *j*-th location among unique locations of all the confirmed cases; *K*_h_(*S* – *L*_*j*_) denotes a Gaussian kernel between locations *S* and *L*_*j*_:

4 $$K{\mathrm{h}}( {S - L_j} ) = \frac{{\mathrm{1}}}{{{\mathrm{2}}\pi \sqrt {\det \left( h \right)} }}\exp \left( { - \frac{1}{2}( {S - L_j} )^Th^{ - 1}( {S - L_j} )} \right)$$

where h denotes the bandwidth matrix equating to $n^{^{ - 1/3}}\hat {\Sigma}$, with *n* being the total number of confirmed cases in all 82 days, and $\hat {\Sigma}$ being the covariance matrix of (*x*_1_, …, *x*_*n*_) and (*y*_1_ …, *y*_*n*_), denoting the vectors of *x* and *y* coordinates of *L*_1_, *L*_2_,… *L*_*n*_. *M*(*S*, *t*_*i*_) denotes a human mobility factor at location *S*, on day *t*_*i*_, calculated as:

5 $$M\left( {S,t_i} \right) = \left\{ {\begin{array}{*{20}{c}} {i^{ - 1}\mathop {\sum}\nolimits_{k = 1}^i {b_{Sk}V_k,} } & {S\,{\mathrm{is}}\,{\mathrm{outside}}\,{\mathrm{Wuhan}}} \\ {1,} \hfill& {S\,{\mathrm{is}}\,{\mathrm{inside}}\,{\mathrm{Wuhan}}} \end{array}} \right.$$

where *b*_*Sk*_ denotes the daily percentage of Wuhan migration to the city containing location *S*, on day *t*_*k*_; *V*_*k*_ denotes the daily Wuhan out-migration to other cities on day *t*_*k*_ prior to *t*_*i*_. Note that *M*(*S*, *t*_*i*_) after the lockdown day *t*_47_ is calculated differently under two scenarios, i.e., with and without the Wuhan lockdown having been implemented. *M*(*S*, *t*_*i*_) after *t*_47_ is equal to *M*(*S*, *t*_47_) under the lockdown scenario. This was due to: (a) very small flows of humans and vehicles leaving Wuhan after the lockdown. Also, such journeys were mostly for critical needs, such as medical treatment; (b) strict quarantine measures imposed on these outflows^9^. Under the non-lockdown scenario, after *t*_47_, *b*_*Sk*_ and *V*_*k*_ values on the same lunar calendar date in 2019 as *t*_*k*_ are assigned to *b*_*Sk*_ and *V*_*k*_, since the migration patterns were similar during that period over two years^11^. Then *M*(*S*, *t*_*i*_) after *t*_47_ is computed accordingly.

*Step 3*: to predict the risk of COVID-19 onset at each random location on a specific day in near future:

6 $$P_{{\mathrm{onset}}}\left( {S,t_z} \right) = 1 - \mathop {\prod}\limits_{t_i < t_z} {\left( {1 - P_{{\mathrm{infection}}}\left( {S,t_i} \right)p_{{\mathrm{incubation}}}\left( {t_z - t_i} \right)} \right)}$$

where *P*_onset_(*S*, *t*_*z*_) denotes the likelihood that at least one person infected by a confirmed case at location *S* develops clinical symptoms on day *t*_*z*_; *t*_*i*_ denotes the date of infection for that person, so always *i* < *z*. Note that *P*_onset_(*S*, *t*_*z*_) values represent point estimates over the continuous space. Such a risk measure may not be intuitive for decision making, and hence could be standardised to the range of 0 and 1 by being divided by the maximum predicted risk among all locations on day *t*_*z*_. By doing so, the standardised predicted risk is seen as the relative risk of symptom onset to the highest risk of symptom onset in the study area. Hence, it can serve as an intuitive indicator suitable for epidemic control and also be a strong alert that prevention work must begin. Estimates of the risk can be averaged flexibly in any areal unit such as a city or residential community, and hence could overcome the modifiable areal unit problem (MAUP) during epidemic response^48^.

### Assessment of prediction models

The accuracy of the predicted risk of symptom onset was evaluated daily, by calculating the percentage of confirmed cases reported in areas where the predicted symptom onset risk was >0.8. The predicted results were also compared to results from the original WKDE model^24^. Since prediction errors could accumulate with time as regards a prediction for further future, the risk predicted on the basis of data no later than “the day before” is usually the most accurate. As a consequence, all risks of symptom onset, mentioned in this study, have been predicted based on confirmed COVID-19 cases with onset dates no later than the previous day. However, the risk on 27 February 2020 was predicted based on the data on or before 20 February 2020 (the final date of symptom onset estimated from confirmed cases).

### Statistics and reproducibility

Statistical significance of data was tested by two-sample *T*-test and/or the Wilcoxon signed-rank test by using Excel and SPSS (https://www.spss-tutorials.com/spss-wilcoxon-signed-ranks-test-simple-example/), respectively. The sample size (*n*) and the nature of replicates have been given wherever relevant.

### Reporting summary

Further information on research design is available in the Nature Research Reporting Summary linked to this article.

## Supplementary information

Supplementary Information

Description of Additional Supplementary Files

Supplementary Data 1

Supplementary Data 2

Supplementary Data 3

Supplementary Data 4

Supplementary Data 5

Reporting Summary

## Data Availability

The data generated or analysed during this study are included in this published article and related Supplementary files (Supplementary Data 1–5) or are available upon reasonable request.

## Code availability

The MATLAB scripts developed and used in this study are available at the DOI-minting repository (Zenodo)^49^.
