# Supplementary material for: An extended Weight Kernel Density Estimation model forecasts COVID-19 onset risk and identifies spatiotemporal variations of lockdown effects in China
Source: Commun Biol. 2021 Jan 25;4:126. doi: 10.1038/s42003-021-01677-2 (PMC7835364; doi:10.1038/s42003-021-01677-2)
Supplement: Supplementary file 1 — Supplementary Information [file 42003_2021_1677_MOESM1_ESM.pdf]

An extended Weight Kernel Density Estimation model forecasts COVID-19 onset risk and identifies spatiotemporal variations of lockdown effects in China

- Supplementary Information

Wenzhong Shi, Chengzhuo Tong, Anshu Zhang, Bin Wang, Zhicheng Shi, Yepeng Yao, Peng Jia

Within this PDF:

Supplementary Table 1. Selection criteria for the seven cities

Supplementary Table 1. Selection criteria for the seven cities. These cities were selected to make them representative in terms of population, GDP, spatial distance to Wuhan, the percentage of migrants from Wuhan before the lockdown date 23rd January, and China's official city-size classification.

	City	Urban population	GDP (Trillion CNY)	Geographical distance from Wuhan (Km)	% of migrants from Wuhan before 23rd January 2020	City-size Classification[1]
1	Shanghai	24,237,800	3.82	833	0.25%	Mega-size
2	Beijing	18,634,000	3.50	1,179	0.25%	Mega-size
3	Shenzhen	13,026,600	2.60	1,055	0.40%	Mega-size
4	Luoyang	2,067,000	0.50	571	0.04%	Major-size
5	Zhangzhou	817,700	0.31	968	0.02%	Medium-size
6	Xiangtan	788,400	0.22	430	0.06%	Medium-size
7	Hanzhong	490,400	0.15	916	0.02%	Minor-size

References

1.Wei, Q. I., Shenghe, L. & Haoran, J. Applicability of the new standard of city-size classification in China. *Prog. Hum. Geogr.* **35**, 47-56 (2016).
