# Supplementary material for: An extended Weight Kernel Density Estimation model forecasts COVID-19 onset risk and identifies spatiotemporal variations of lockdown effects in China
Source: Commun Biol. 2021 Jan 25;4:126. doi: 10.1038/s42003-021-01677-2 (PMC7835364; doi:10.1038/s42003-021-01677-2)
Supplement: Supplementary file 2 — Description of Additional Supplementary Files [file 42003_2021_1677_MOESM2_ESM.pdf]

Description of Additional Supplementary Items

File name: Supplementary Data 1

Description: This file contains the data about 1-14 days accuracy of the predicted risk of COVID-19 symptom onset by the extended and original Weight Kernel Density Estimation (WKDE) models.

File name: Supplementary Data 2

Description: This file contains the data about the risk of COVID-19 symptom onset under two scenarios (i.e., with and without Wuhan lockdown measure) on 25 Jan 2020, 30 Jan 2020, and 5 Feb 2020.

File name: Supplementary Data 3

Description: This file contains the data about the risk of COVID-19 symptom onset under two scenarios (i.e., with and without Wuhan lockdown measure) from 24th January to 5th February 2020 in Shanghai, Beijing, Shenzhen, Luoyang (in Henan Province), Xiangtan (Hunan), Zhangzhou (Fujian), and Hanzhong (Shaanxi).

File name: Supplementary Data 4

Description: This file contains the data about Distribution of the estimated dates of COVID-19 symptom onset among 40,486 confirmed cases used in this study, and of the actual dates of COVID-19 symptom onset among 75,465 confirmed cases reported by the World Health Organization.

File name: Supplementary Data 5

Description: This file contains the data about data source websites of confirmed cases in 347 cities from official reports and public media.
