# Supplementary material for: An extended Weight Kernel Density Estimation model forecasts COVID-19 onset risk and identifies spatiotemporal variations of lockdown effects in China
Source: Commun Biol. 2021 Jan 25;4:126. doi: 10.1038/s42003-021-01677-2 (PMC7835364; doi:10.1038/s42003-021-01677-2)
Supplement: Supplementary file 8 — Reporting Summary [file 42003_2021_1677_MOESM8_ESM.pdf]

nature research

Corresponding author(s): Wenzhong Shi
Last updated by author(s): Nov 23, 2020

Reporting Summary

Nature Research wishes to improve the reproducibility of the work that we publish. This form provides structure for consistency and transparency in reporting. For further information on Nature Research policies, see our Editorial Policies and the Editorial Policy Checklist.

Statistics

For all statistical analyses, confirm that the following items are present in the figure legend, table legend, main text, or Methods section.

n/a	Confirmed	
☐	☒	The exact sample size (*n*) for each experimental group/condition, given as a discrete number and unit of measurement
☒	☐	A statement on whether measurements were taken from distinct samples or whether the same sample was measured repeatedly
☒	☐	The statistical test(s) used AND whether they are one- or two-sided<br>*Only common tests should be described solely by name; describe more complex techniques in the Methods section.*
☒	☐	A description of all covariates tested
☒	☐	A description of any assumptions or corrections, such as tests of normality and adjustment for multiple comparisons
☐	☒	A full description of the statistical parameters including central tendency (e.g. means) or other basic estimates (e.g. regression coefficient) AND variation (e.g. standard deviation) or associated estimates of uncertainty (e.g. confidence intervals)
☒	☐	For null hypothesis testing, the test statistic (e.g. *F*, *t*, *r*) with confidence intervals, effect sizes, degrees of freedom and *P* value noted<br>*Give P values as exact values whenever suitable.*
☒	☐	For Bayesian analysis, information on the choice of priors and Markov chain Monte Carlo settings
☒	☐	For hierarchical and complex designs, identification of the appropriate level for tests and full reporting of outcomes
☒	☐	Estimates of effect sizes (e.g. Cohen's *d*, Pearson's *r*), indicating how they were calculated

Our web collection on statistics for biologists contains articles on many of the points above.

Software and code

Policy information about availability of computer code

Data collection	No software was used for data collection.
Data analysis	Custom Matlab code was used to implement the original and extended WKDE models for predicting the risk of COVID-19 onset used in this study.

For manuscripts utilizing custom algorithms or software that are central to the research but not yet described in published literature, software must be made available to editors and reviewers. We strongly encourage code deposition in a community repository (e.g. GitHub). See the Nature Research guidelines for submitting code & software for further information.

Data

Policy information about availability of data

All manuscripts must include a data availability statement. This statement should provide the following information, where applicable:

- Accession codes, unique identifiers, or web links for publicly available datasets
- A list of figures that have associated raw data
- A description of any restrictions on data availability

The data that support the findings of this study are included in the paper and its supplementary information files or available from the corresponding author upon request.

Field-specific reporting

Please select the one below that is the best fit for your research. If you are not sure, read the appropriate sections before making your selection.

☐ Life sciences ☒ Behavioural & social sciences ☐ Ecological, evolutionary & environmental sciences

For a reference copy of the document with all sections, see nature.com/documents/nr-reporting-summary-flat.pdf

Behavioural & social sciences study design

All studies must disclose on these points even when the disclosure is negative.

Study description	This is a quantitative study. We forecast the risk of COVID-19 symptom onset by using public available data on confirmed cases in China with locations in residential community level and public data on human mobility. Then we evaluated the effects of the Wuhan lockdown on the risk of the COVID-19 onset based on the forecasting result.
Research sample	This study used all the 40,486 confirmed cases in China with available community-level locations where they had a period of stay prior to diagnosis, among a total of 75,465 confirmed cases in China during the study period reported by the World Health Organization (WHO; the WHO report did not contain detailed locations of the cases needed by this study). The daily migration data from Baidu were the total migration of all people from Wuhan to every other city, as estimated by Baidu from the massive positioning service data of the company.
Sampling strategy	All available data for confirmed cases with community-level locations (N=40,486) were used for predicting the COVID-19 onset risk on future dates, without sampling.
Data collection	The data for confirmed cases with community-level locations, as well as the human mobility data (including the migration data from Baidu Map and daily passenger loads of flights, railways and bus services) were collected manually from the public available online sources.
Timing	Data on confirmed COVID-19 cases in China were collected from the 31st December 2019 to 2nd March 2020. To obtain the human mobility during the study period and estimate the human mobility if the Wuhan lockdown measure had not been imposed, the daily migration data from Baidu were collected during January-March 2019 and 2020, and daily passenger loads of all flights, railways and bus services from Wuhan to every other city were collected from December 2019 to March 2020.
Data exclusions	No data of the collected COVID-19 confirmed cases were excluded. The stated 40,486 cases were those with complete information needed by this study and were all used for forecasting future onset risks.
Non-participation	The COVID-19 case data and human mobility data used in this study were collected from publicly available data sources, without a problem of non-participation.
Randomization	The study is not a group experiment, so the participants did not need to be allocated into groups.

Reporting for specific materials, systems and methods

We require information from authors about some types of materials, experimental systems and methods used in many studies. Here, indicate whether each material, system or method listed is relevant to your study. If you are not sure if a list item applies to your research, read the appropriate section before selecting a response.

Materials & experimental systems

n/a	Involved in the study	
☒	☐	Antibodies
☒	☐	Eukaryotic cell lines
☒	☐	Palaeontology and archaeology
☒	☐	Animals and other organisms
☒	☐	Human research participants
☒	☐	Clinical data
☒	☐	Dual use research of concern

Methods

n/a	Involved in the study	
☒	☐	ChIP-seq
☒	☐	Flow cytometry
☒	☐	MRI-based neuroimaging
